# Supplementary material for: Using a Stifneck Select CollarTM for hands-free semiautomatic blood flow measurements: A user study
Source: PLoS One. 2026 Sep 23;21(9):e0358589. doi: 10.1371/journal.pone.0358589 (PMC13600611; doi:10.1371/journal.pone.0358589)
Supplement: Supplementary file 1 [file pone.0358589.s003.docx]

**Response to Reviewers**

We thank the reviewers for their assessments and their supportive notes and questions. In the following, we address their concerns point by point.

Reviewer #1:

Issue / Concern # 1:

The demographic data indicate a large variability (e.g., age 19 – 83 years, BMI 34.4, etc.). It would be interesting to know whether physiological variables such as gender/body weight/neck circumference have any impact on the doppler flow values. E.g., by stratifying the groups at the variable's median, the measurements could be described separately for each group. Alternatively, the mean/median Doppler flow values could also be analyzed in relation to the above-mentioned parameters by regression/correlation analysis.

Author response:

We thank the reviewer for this question and are happy to announce that this analysis is part of a future publication. The usage of the Stifneck Select Collar™ was part of a larger study, which aims at researching, among other questions, the relation between patient properties and valid sensor placement. Due to the many aspects of the study, its results are divided and published iteratively to allow concise evaluations that build on peer-reviewed methods. The feasibility of the neck brace mount is one of the first steps. Results of the relation between physiological variables and Doppler flow values will be the subject of future publications and conference poster demonstrations. Due to the amount of data, we felt it best to divide the scientific questions and treat single aspects separately.

Author action:

n.a.

Reviewer #2:

Issue / Concern # 1: Introduction

No Flow Time usually refers to the period when no CPR is performed, not even by non-medical personnel. This should be clearly mentioned since it cannot be addressed by the presented device.

Author response:

We gladly specify the differentiation and also add more discussion points on recognizing low flow times during CPR, which lack precise flow volume thresholds due to the state‑of‑the‑art.

Author action:

in section Introduction

The survival rates of out‑of‑hospital cardiac arrest (OHCA) remain low, ranging from 0% to 15% at the time of hospital discharge [1]. Long *no flow time* (NFT), i.e., periods of time where no cardiopulmonary resuscitation (CPR) is performed, has been identified as one of the crucial factors influencing long-term hospital outcomes after OHCA. *Low flow time* (LFT), i.e., periods of time where CPR results in small blood flow volumes, has a similar effect, albeit its exact correlation to medical outcome is still subject to research.

in section Discussion – Limitations and future works

The system is also limited in the way that it can not approximate the entire NFT period duration, since NFT periods can occur before system application. The automatic identification of LFT periods is yet to be researched, due to the fact that there is no scientific consensus that defines what amount of flow volume constitutes as sufficient or too low for effective CPR. Further studies have to be conducted to analyze the blood flow volumes of multiple CPR efforts and the respective long‑term medical outcome.

Issue / Concern # 2: Materials and Methods

Can the device be mounted on any standard neck brace, or does it need to be prefabricated? This is important to me because I want to know if it is necessary to remove an already positioned stiff neck if the patient suffers cardiac arrest during transport (e.g., trauma patient), as this could potentially put the patient at additional risk.

Author response:

At the moment, the device can be mounted on any already procured standard Stifneck Select Collar™ neck brace. The attachment can also be done without removing the neck brace from the patient. However, placement of the sensor at anterior positions might be restricted when used on patients with larger neck circumferences. This will most likely be an issue when the neck brace attachment itself is already difficult. We aim to address this problem in future developments with simplified, alternative neck brace solutions.

Author action:

in section Discussion – The Construction

The sensor mount can be attached to any already procured standard Stifneck Select Collar™ neck brace. The attachment can also be done without removing the neck brace from the patient.

Issue / Concern # 3: Materials and Methods

How easy is it to place the collar and the probe, including the time from start to the first measurement? I understand this is a preliminary report under test conditions, but could you estimate how difficult and time-consuming it might be under stress or CPR conditions?

Author response:

As mentioned by the reviewer, during our proband study we faced test conditions; we therefore prioritized comfort and the prevention of possible harm. Periodic communication with our volunteers, purposeful slow and careful adjustment, and slight adjustments to optimize comfort extended the actual application time. In the end, application and interview time was less than a minute. Analysis in real scenario studies will follow, evaluating application time and also proficiency when the system is used by differently trained people.

We estimate that future versions of our system could allow for an application time that is as difficult and time‑consuming as a normal Stifneck Select Collar™ attachment under CPR conditions. Shortest durations could therefore be as short as ten to twenty seconds, depending on the patient’s condition, after which measurements could be initiated immediately. This will require a trained user, a smaller version of our system, and a standard sensor position which guarantees sensor-vessel alignment. Our current efforts address the two latter issues.

Author action:

in section Methods and Materials - Scaled assessments

During our study, volunteer comfort and avoidance of harm were prioritized. Communication and purposefully slow system application increased setup time. Durations that represent only Stifneck Select Collar™ attachment were not recorded.

in section Results

Following training in the correct handling and application of the Stifneck Select Collar™, overall application duration in the study was generally below one minute in healthy participants.

in section Discussion - Limitations and future works

The system placement usability in emergency scenarios, i.e., the fastest possible setup without causing harm, has not been addressed in this work either and needs to be analyzed further. At the moment, the device is applied during interviews with volunteers and with a priority on comfort and the prevention of harm, which increases the duration of positioning time and prevents conclusive statements about the fastest valid application time. Without the interview process and with a standard sensor position that could provide high sensor vessel alignment probability, the time to start measurements would decrease significantly from the current value of one minute. System application and measurement initiation times could then hypothetically be reduced to the standard duration of Stifneck Select Collar™ fixation, when used by a proficient user.

Issue / Concern # 4: Materials and Methods

The figure of the collar indicates that intubation or assisted ventilation might be affected during the placement of the collar. Please write one sentence about this potential problem.

Author response:

We thank the reviewer for raising this important issue. Cervical collars are routinely used in pre-hospital polytrauma management when a cervical spine injury is suspected. Manual in-line stabilization of the cervical spine is recommended during endotracheal intubation, after which a cervical collar can be applied to maintain immobilization of the cervical spine. Although cervical collars may affect airway management and ventilation, we anticipate this effect to be minimal when the collar is applied appropriately and removed during intubation. Easy reattachment is made possible due to the hook-and-loop system of the collar, while the fixed sensor mount should reproduce a similar, if not identical, sensor position.

Author action:

in section Discussion - Limitations and future works

In a real resuscitation scenario, the cervical collar also needs to be temporarily removed during endotracheal intubation, as it may impede airway management and interfere with the intubation procedure. This is quickly achieved with the hook-and-loop system of the collar, keeping intubation delays to a minimum. After airway control has been established, it can be reapplied quickly, while the fixed sensor mount guarantees an almost identical sensor position as before detachment.

Issue / Concern # 5: Discussion

While all devices that enhance CPR effectiveness are valuable, the most important are those that can monitor CPR efficacy beyond just pupil size or PaCO2.

Given that rapid, continuous CPR is crucial, the authors should discuss how quickly and easily the device can be positioned during CPR, and whether the process of intubation or assisted ventilation might be affected or delayed by placing the device.

Author response:

We are happy to improve our manuscript in that regard and are grateful for the reviewer to have pointed out that missing discussion content. In line with concerns #4 and #5, we provided the additional discussions in our manuscript.

Author action:

in section Discussion - Limitations and future works

The system placement usability in emergency scenarios, i.e., the fastest possible setup without causing harm, has not been addressed in this work either and needs to be analyzed further. At the moment, the device is applied during interviews with volunteers and with a priority on comfort and the prevention of harm, which increases the duration of positioning time and prevents conclusive statements about the fastest valid application time. Without the interview process and with a standard sensor position that could provide high sensor vessel alignment probability, the time to start measurements would decrease significantly from the current value of one minute. System application and measurement initiation times could then hypothetically be reduced to the standard duration of Stifneck Select Collar™ fixation, when used by a proficient user.

In a real resuscitation scenario, the cervical collar also needs to be temporarily removed during endotracheal intubation, as it may impede airway management and interfere with the intubation procedure. This is quickly achieved with the hook-and-loop system of the collar, keeping intubation delays to a minimum. After airway control has been established, it can be reapplied quickly, while the fixed sensor mount guarantees an almost identical sensor position as before detachment.

Issue / Concern # 6: Discussion

Furthermore, blood flow in the CCA does not always indicate adequate brain perfusion. Elevated ICP from bleeding, edema, or similar causes may impair blood flow to the brain even if CCA values are normal. Since severe hypoxic brain damage is the most common cause of adverse outcomes following CPR, this important point should be briefly noted.

Author response:

We added the relevant information in a short text to our discussion and are grateful for this valuable note. Since the state‑of‑the‑art does not provide a large database of Doppler measurements taken during cardiopulmonary resuscitation, we aim to gather a dedicated database in order to research the aforementioned problems.

Author action:

in section Discussion - Limitations and future works

Data collection and analysis also have to include cases with impaired cerebral perfusion. While measured values during CPR might indicate an effective blood flow through the CCA, injuries or sicknesses can still prohibit the actual brain perfusion. Any system feedback about sufficient blood flow might mislead the user about the therapy efficacy and result in severe hypoxic brain damage. At the current moment, the system cannot recognize these problems. Further research and development with a dedicated database is necessary to evaluate if and how the system can gather indications about cerebral flow issues.
