# Supplementary material for: Using a Stifneck Select CollarTM for hands-free semiautomatic blood flow measurements: A user study
Source: PLoS One. 2026 Sep 23;21(9):e0358589. doi: 10.1371/journal.pone.0358589 (PMC13600611; doi:10.1371/journal.pone.0358589)
Supplement: Peer review history

# Peer review: Decision Letter 0

**DOI:** 10.1371/journal.pone.0358589.r001

**Authors:** Martin Kieninger

4 Aug 2026

Dear Dr. Fuchs,

Thank you for submitting your manuscript to PLOS ONE. After careful consideration, we feel that it has merit but does not fully meet PLOS ONE’s publication criteria as it currently stands. Therefore, we invite you to submit a revised version of the manuscript that addresses the points raised during the review process.

Please submit your revised manuscript by Sep 18 2026 11:59PM. If you will need more time than this to complete your revisions, please reply to this message or contact the journal office at plosone@plos.org. When you're ready to submit your revision, log on to https://www.editorialmanager.com/pone/ and select the 'Submissions Needing Revision' folder to locate your manuscript file.

- A letter that responds to each point raised by the academic editor and reviewer(s). You should upload this letter as a separate file labeled 'Response to Reviewers'.
- A marked-up copy of your manuscript that highlights changes made to the original version. You should upload this as a separate file labeled 'Revised Manuscript with Track Changes'.
- An unmarked version of your revised paper without tracked changes. You should upload this as a separate file labeled 'Manuscript'.

If you would like to make changes to your financial disclosure, please include your updated statement in your cover letter. Guidelines for resubmitting your figure files are available below the reviewer comments at the end of this letter.

If applicable, we recommend that you deposit your laboratory protocols in protocols.io to enhance the reproducibility of your results. Protocols.io assigns your protocol its own identifier (DOI) so that it can be cited independently in the future. For instructions see: https://journals.plos.org/plosone/s/submission-guidelines#loc-laboratory-protocols. Additionally, PLOS ONE offers an option for publishing peer-reviewed Lab Protocol articles, which describe protocols hosted on protocols.io. Read more information on sharing protocols at https://plos.org/protocols?utm_medium=editorial-email&utm_source=authorletters&utm_campaign=protocols.

As the corresponding author, your ORCID iD is verified in the submission system and will appear in the published article. PLOS supports the use of ORCID, and we encourage all coauthors to register for an ORCID iD and use it as well. Please encourage your coauthors to verify their ORCID iD within the submission system before final acceptance, as unverified ORCID iDs will not appear in the published article. *Only*  the individual author can complete the verification step; PLOS staff *cannot*  verify ORCID iDs on behalf of authors.

We look forward to receiving your revised manuscript.

Kind regards,

Martin Kieninger

Academic Editor

PLOS One

Journal requirements:

When submitting your revision, we need you to address these additional requirements.

1. Please ensure that your manuscript meets PLOS ONE's style requirements, including those for file naming. The PLOS ONE style templates can be found at

https://journals.plos.org/plosone/s/file?id=wjVg/PLOSOne_formatting_sample_main_body.pdf and

https://journals.plos.org/plosone/s/file?id=ba62/PLOSOne_formatting_sample_title_authors_affiliations.pdf

2. Thank you for stating the following in the Financial Disclosure section:

“The project was supported by grants from the German Federal Ministry for Economic Affairs and Energy (Grant No. 16KN083328), the Deutsche Herzstiftung e.V., Frankfurt a. M., and the German Federal Ministry of Research, Technology, and Space (Grant No. 13GW0768B). The publication was supported by the Open Access Publishing Fund of Leipzig University. The funders had no role in study design, data collection and analysis, decision to publish, or preparation of the manuscript.”

We note that one or more of the authors are employed by a commercial company: GAMPT mbH.

1.        Please provide an amended Funding Statement declaring this commercial affiliation, as well as a statement regarding the Role of Funders in your study. If the funding organization did not play a role in the study design, data collection and analysis, decision to publish, or preparation of the manuscript and only provided financial support in the form of authors' salaries and/or research materials, please review your statements relating to the author contributions, and ensure you have specifically and accurately indicated the role(s) that these authors had in your study. You can update author roles in the Author Contributions section of the online submission form.

Please also include the following statement within your amended Funding Statement.

“The funder provided support in the form of salaries for authors [insert relevant initials], but did not have any additional role in the study design, data collection and analysis, decision to publish, or preparation of the manuscript. The specific roles of these authors are articulated in the ‘author contributions’ section.”

If your commercial affiliation did play a role in your study, please state and explain this role within your updated Funding Statement.

2. Please also provide an updated Competing Interests Statement declaring this commercial affiliation along with any other relevant declarations relating to employment, consultancy, patents, products in development, or marketed products, etc.

Within your Competing Interests Statement, please confirm that this commercial affiliation does not alter your adherence to all PLOS ONE policies on sharing data and materials by including the following statement: "This does not alter our adherence to  PLOS ONE policies on sharing data and materials.” (as detailed online in our guide for authors http://journals.plos.org/plosone/s/competing-interests) . If this adherence statement is not accurate and  there are restrictions on sharing of data and/or materials, please state these. Please note that we cannot proceed with consideration of your article until this information has been declared.

Please include both an updated Funding Statement and Competing Interests Statement in your cover letter. We will change the online submission form on your behalf.

3. We note that Figures 1 and 2 in your submission contain copyrighted images. All PLOS content is published under the Creative Commons Attribution License (CC BY 4.0), which means that the manuscript, images, and Supporting Information files will be freely available online, and any third party is permitted to access, download, copy, distribute, and use these materials in any way, even commercially, with proper attribution. For more information, see our copyright guidelines: http://journals.plos.org/plosone/s/licenses-and-copyright.

We require you to either (1) present written permission from the copyright holder to publish these figures specifically under the CC BY 4.0 license, or (2) remove the figures from your submission:

1. You may seek permission from the original copyright holder of Figures 1 and 2 to publish the content specifically under the CC BY 4.0 license.

We recommend that you contact the original copyright holder with the Content Permission Form (http://journals.plos.org/plosone/s/file?id=7c09/content-permission-form.pdf) and the following text:

“I request permission for the open-access journal PLOS ONE to publish XXX under the Creative Commons Attribution License (CCAL) CC BY 4.0 (http://creativecommons.org/licenses/by/4.0/). Please be aware that this license allows unrestricted use and distribution, even commercially, by third parties. Please reply and provide explicit written permission to publish XXX under a CC BY license and complete the attached form.”

Please upload the completed Content Permission Form or other proof of granted permissions as an "Other" file with your submission.

In the figure caption of the copyrighted figure, please include the following text: “Reprinted from [ref] under a CC BY license, with permission from [name of publisher], original copyright [original copyright year].”

2. If you are unable to obtain permission from the original copyright holder to publish these figures under the CC BY 4.0 license or if the copyright holder’s requirements are incompatible with the CC BY 4.0 license, please either i) remove the figure or ii) supply a replacement figure that complies with the CC BY 4.0 license. Please check copyright information on all replacement figures and update the figure caption with source information. If applicable, please specify in the figure caption text when a figure is similar but not identical to the original image and is therefore for illustrative purposes only.

If the reviewer comments include a recommendation to cite specific previously published works, please review and evaluate these publications to determine whether they are relevant and should be cited. There is no requirement to cite these works unless the editor has indicated otherwise.

Please review your reference list to ensure that it is complete and correct. If you have cited papers that have been retracted, please include the rationale for doing so in the manuscript text, or remove these references and replace them with relevant current references. Any changes to the reference list should be mentioned in the rebuttal letter that accompanies your revised manuscript. If you need to cite a retracted article, indicate the article’s retracted status in the References list and also include a citation and full reference for the retraction notice.

[Note: HTML markup is below. Please do not edit.]

Reviewers' comments:

Reviewer's Responses to Questions

**Comments to the Author**

1. Is the manuscript technically sound, and do the data support the conclusions?

Reviewer #1: Yes

Reviewer #2: Yes

**********

2. Has the statistical analysis been performed appropriately and rigorously?

Reviewer #1: Yes

Reviewer #2: Yes

**********

3. Have the authors made all data underlying the findings in their manuscript fully available?

The PLOS Data policy

Reviewer #1: Yes

Reviewer #2: Yes

**********

4. Is the manuscript presented in an intelligible fashion and written in standard English?

Reviewer #1: Yes

Reviewer #2: Yes

**********

Reviewer #1: The authors describe an innovative method for monitoring the efficacy of CPR with regard to cerebral perfusion by way of a customized stiffneck holding ultrasound probes in place. The study was conducted in 102 volunteers; no significant pain / dyscomfort was reported.

This is an interesting development that addresses the potentially insufficient cerebral blood flow / oxygen transport during CPR, reflecting a well-known unmet medical need. Since such a device is highly desirable in clinical practice, both pre- and in-hospital, this descriptive feasibility study undoubtedly warrants publication.

There is only one aspect I would be interested in: The demographic data indicate a large variability (e.g., age 19 – 83 years, BMI 34.4, etc.). It would be interesting to know whether physiological variables such as gender/body weight/neck circumference have any impact on the doppler flow values. E.g., by stratifying the groups at the variable's median, the measurements could be described separately for each group. Alternatively, the mean/median Doppler flow values could also be analyzed in relation to the above-mentioned parameters by regression/correlation analysis.

Reviewer #2: In this paper, the authors describe an advanced device using a neck collar to hands-free and continuously measure blood flow in the CCA. The future goal is to use this device during CPR to assess the effectiveness of the performed CPR in individual cases.

This is a very important question during CPR that is not sufficiently addressed in current research, despite studies using adhesives to attach Doppler probes and neck braces. So far, the success and effectiveness of such devices remain unclear because they are often tricky to place and frequently dislocate during CPR or similar body movements. The introduced model appears promising, as do the results of this preliminary study.

Due to the need of improvements in this field, this paper is significant, and because of the presented feasibility of the new arranged device, this paper should be published.

But there are some points which need to be adressed by the authors in my opinion.

Introduction:

No Flow Time usually refers to the period when no CPR is performed, not even by non-medical personnel. This should be clearly mentioned since it cannot be addressed by the presented device.

Materials and Methods:

Can the device be mounted on any standard neck brace, or does it need to be prefabricated? This is important to me because I want to know if it is necessary to remove an already positioned stiff neck if the patient suffers cardiac arrest during transport (e.g., trauma patient), as this could potentially put the patient at additional risk.

How easy is it to place the collar and the probe, including the time from start to the first measurement? I understand this is a preliminary report under test conditions, but could you estimate how difficult and time-consuming it might be under stress or CPR conditions?

The figure of the collar indicates that intubation or assisted ventilation might be affected during the placement of the collar. Please write one sentence about this potential problem.

Results:

The results are well presented and understandable. The collar does not seem to cause any harm (such as skin lesions) and appears wearable in awake and healthy volunteers, which is a significant advantage of the device compared to adhesives.

Discussion:

While all devices that enhance CPR effectiveness are valuable, the most important are those that can monitor CPR efficacy beyond just pupil size or PaCO2.

Given that rapid, continuous CPR is crucial, the authors should discuss how quickly and easily the device can be positioned during CPR, and whether the process of intubation or assisted ventilation might be affected or delayed by placing the device.

Furthermore, blood flow in the CCA does not always indicate adequate brain perfusion. Elevated ICP from bleeding, edema, or similar causes may impair blood flow to the brain even if CCA values are normal. Since severe hypoxic brain damage is the most common cause of adverse outcomes following CPR, this important point should be briefly noted.

Since this is a preliminary dataset collected under study conditions with healthy volunteers, my comments may seem quite critical. However, these are essential questions for researchers in the field. I would appreciate it if the authors could address at least some of these points in their discussion.

**********

what does this mean?). If published, this will include your full peer review and any attached files.

If you choose “no”, your identity will remain anonymous but your review may still be made public.

**Do you want your identity to be public for this peer review?**  For information about this choice, including consent withdrawal, please see our Privacy Policy

Reviewer #1: No

Reviewer #2: No

**********

[NOTE: If reviewer comments were submitted as an attachment file, they will be attached to this email and accessible via the submission site. Please log into your account, locate the manuscript record, and check for the action link "View Attachments". If this link does not appear, there are no attachment files.]

To ensure your figures meet our technical requirements, please review our figure guidelines: https://journals.plos.org/plosone/s/figures

You may also use PLOS’s free figure tool, NAAS, to help you prepare publication quality figures: https://journals.plos.org/plosone/s/figures#loc-tools-for-figure-preparation.

NAAS will assess whether your figures meet our technical requirements by comparing each figure against our figure specifications.

# Author comment: Author response to Decision Letter 1

**DOI:** 10.1371/journal.pone.0358589.r002

24 Aug 2026

We thank the reviewers for their assessments and their supportive notes and questions. In the following, we address their concerns point by point.

Reviewer #1:

Issue / Concern # 1:

The demographic data indicate a large variability (e.g., age 19 – 83 years, BMI 34.4, etc.). It would be interesting to know whether physiological variables such as gender/body weight/neck circumference have any impact on the doppler flow values. E.g., by stratifying the groups at the variable's median, the measurements could be described separately for each group. Alternatively, the mean/median Doppler flow values could also be analyzed in relation to the above-mentioned parameters by regression/correlation analysis.

Author response:

We thank the reviewer for this question and are happy to announce that this analysis is part of a future publication. The usage of the Stifneck Select Collar™ was part of a larger study, which aims at researching, among other questions, the relation between patient properties and valid sensor placement. Due to the many aspects of the study, its results are divided and published iteratively to allow concise evaluations that build on peer-reviewed methods. The feasibility of the neck brace mount is one of the first steps. Results of the relation between physiological variables and Doppler flow values will be the subject of future publications and conference poster demonstrations. Due to the amount of data, we felt it best to divide the scientific questions and treat single aspects separately.

Author action:

n.a.

________________________________________

Reviewer #2:

Issue / Concern # 1: Introduction

No Flow Time usually refers to the period when no CPR is performed, not even by non-medical personnel. This should be clearly mentioned since it cannot be addressed by the presented device.

Author response:

We gladly specify the differentiation and also add more discussion points on recognizing low flow times during CPR, which lack precise flow volume thresholds due to the state of the art.

Author action:

in section Introduction

The survival rates of out of hospital cardiac arrest (OHCA) remain low, ranging from 0% to 15% at the time of hospital discharge [1]. Long no flow time (NFT), i.e., periods of time where no cardiopulmonary resuscitation (CPR) is performed, has been identified as one of the crucial factors influencing long-term hospital outcomes after OHCA. Low flow time (LFT), i.e., periods of time where CPR results in small blood flow volumes, has a similar effect, albeit its exact correlation to medical outcome is still subject to research.

in section Discussion – Limitations and future works

The system is also limited in the way that it can not approximate the entire NFT period duration, since NFT periods can occur before system application. The automatic identification of LFT periods is yet to be researched, due to the fact that there is no scientific consensus that defines what amount of flow volume constitutes as sufficient or too low for effective CPR. Further studies have to be conducted to analyze the blood flow volumes of multiple CPR efforts and the respective long term medical outcome.

________________________________________

Issue / Concern # 2: Materials and Methods

Can the device be mounted on any standard neck brace, or does it need to be prefabricated? This is important to me because I want to know if it is necessary to remove an already positioned stiff neck if the patient suffers cardiac arrest during transport (e.g., trauma patient), as this could potentially put the patient at additional risk.

Author response:

At the moment, the device can be mounted on any already procured standard Stifneck Select Collar™ neck brace. The attachment can also be done without removing the neck brace from the patient. However, placement of the sensor at anterior positions might be restricted when used on patients with larger neck circumferences. This will most likely be an issue when the neck brace attachment itself is already difficult. We aim to address this problem in future developments with simplified, alternative neck brace solutions.

Author action:

in section Discussion – The Construction

The sensor mount can be attached to any already procured standard Stifneck Select Collar™ neck brace. The attachment can also be done without removing the neck brace from the patient.

________________________________________

Issue / Concern # 3: Materials and Methods

How easy is it to place the collar and the probe, including the time from start to the first measurement? I understand this is a preliminary report under test conditions, but could you estimate how difficult and time-consuming it might be under stress or CPR conditions?

Author response:

As mentioned by the reviewer, during our proband study we faced test conditions; we therefore prioritized comfort and the prevention of possible harm. Periodic communication with our volunteers, purposeful slow and careful adjustment, and slight adjustments to optimize comfort extended the actual application time. In the end, application and interview time was less than a minute. Analysis in real scenario studies will follow, evaluating application time and also proficiency when the system is used by differently trained people.

We estimate that future versions of our system could allow for an application time that is as difficult and time consuming as a normal Stifneck Select Collar™ attachment under CPR conditions. Shortest durations could therefore be as short as ten to twenty seconds, depending on the patient’s condition, after which measurements could be initiated immediately. This will require a trained user, a smaller version of our system, and a standard sensor position which guarantees sensor-vessel alignment. Our current efforts address the two latter issues.

Author action:

in section Methods and Materials - Scaled assessments

During our study, volunteer comfort and avoidance of harm were prioritized. Communication and purposefully slow system application increased setup time. Durations that represent only Stifneck Select Collar™ attachment were not recorded.

in section Results

Following training in the correct handling and application of the Stifneck Select Collar™, overall application duration in the study was generally below one minute in healthy participants.

in section Discussion - Limitations and future works

The system placement usability in emergency scenarios, i.e., the fastest possible setup without causing harm, has not been addressed in this work either and needs to be analyzed further. At the moment, the device is applied during interviews with volunteers and with a priority on comfort and the prevention of harm, which increases the duration of positioning time and prevents conclusive statements about the fastest valid application time. Without the interview process and with a standard sensor position that could provide high sensor vessel alignment probability, the time to start measurements would decrease significantly from the current value of one minute. System application and measurement initiation times could then hypothetically be reduced to the standard duration of Stifneck Select Collar™ fixation, when used by a proficient user.

________________________________________

Issue / Concern # 4: Materials and Methods

The figure of the collar indicates that intubation or assisted ventilation might be affected during the placement of the collar. Please write one sentence about this potential problem.

Author response:

We thank the reviewer for raising this important issue. Cervical collars are routinely used in pre-hospital polytrauma management when a cervical spine injury is suspected. Manual in-line stabilization of the cervical spine is recommended during endotracheal intubation, after which a cervical collar can be applied to maintain immobilization of the cervical spine. Although cervical collars may affect airway management and ventilation, we anticipate this effect to be minimal when the collar is applied appropriately and removed during intubation. Easy reattachment is made possible due to the hook-and-loop system of the collar, while the fixed sensor mount should reproduce a similar, if not identical, sensor position.

Author action:

in section Discussion - Limitations and future works

In a real resuscitation scenario, the cervical collar also needs to be temporarily removed during endotracheal intubation, as it may impede airway management and interfere with the intubation procedure. This is quickly achieved with the hook-and-loop system of the collar, keeping intubation delays to a minimum. After airway control has been established, it can be reapplied quickly, while the fixed sensor mount guarantees an almost identical sensor position as before detachment.

________________________________________

Issue / Concern # 5: Discussion

While all devices that enhance CPR effectiveness are valuable, the most important are those that can monitor CPR efficacy beyond just pupil size or PaCO2.

Given that rapid, continuous CPR is crucial, the authors should discuss how quickly and easily the device can be positioned during CPR, and whether the process of intubation or assisted ventilation might be affected or delayed by placing the device.

Author response:

We are happy to improve our manuscript in that regard and are grateful for the reviewer to have pointed out that missing discussion content. In line with concerns #4 and #5, we provided the additional discussions in our manuscript.

Author action:

in section Discussion - Limitations and future works

The system placement usability in emergency scenarios, i.e., the fastest possible setup without causing harm, has not been addressed in this work either and needs to be analyzed further. At the moment, the device is applied during interviews with volunteers and with a priority on comfort and the prevention of harm, which increases the duration of positioning time and prevents conclusive statements about the fastest valid application time. Without the interview process and with a standard sensor position that could provide high sensor vessel alignment probability, the time to start measurements would decrease significantly from the current value of one minute. System application and measurement initiation times could then hypothetically be reduced to the standard duration of Stifneck Select Collar™ fixation, when used by a proficient user.

In a real resuscitation scenario, the cervical collar also needs to be temporarily removed during endotracheal intubation, as it may impede airway management and interfere with the intubation procedure. This is quickly achieved with the hook-and-loop system of the collar, keeping intubation delays to a minimum. After airway control has been established, it can be reapplied quickly, while the fixed sensor mount guarantees an almost identical sensor position as before detachment.

________________________________________

Issue / Concern # 6: Discussion

Furthermore, blood flow in the CCA does not always indicate adequate brain perfusion. Elevated ICP from bleeding, edema, or similar causes may impair blood flow to the brain even if CCA values are normal. Since severe hypoxic brain damage is the most common cause of adverse outcomes following CPR, this important point should be briefly noted.

Author response:

We added the relevant information in a short text to our discussion and are grateful for this valuable note. Since the state of the art does not provide a large database of Doppler measurements taken during cardiopulmonary resuscitation, we aim to gather a dedicated database in order to research the aforementioned problems.

Author action:

in section Discussion - Limitations and future works

Data collection and analysis also have to include cases with impaired cerebral perfusion. While measured values during CPR might indicate an effective blood flow through the CCA, injuries or sicknesses can still prohibit the actual brain perfusion. Any system feedback about sufficient blood flow might mislead the user about the therapy efficacy and result in severe hypoxic brain damage. At the current moment, the system cannot recognize these problems. Further research and development with a dedicated database is necessary to evaluate if and how the system can gather indications about cerebral flow issues.

________________________________________

Attachment Submitted filename: Response to Reviewers.docx

# Peer review: Decision Letter 1

**DOI:** 10.1371/journal.pone.0358589.r003

**Authors:** Martin Kieninger

2 Sep 2026

Using a Stifneck Select Collar™ for hands-free semiautomatic blood flow measurements: a user study

PONE-D-26-13295R1

Dear Dr. Fuchs,

We’re pleased to inform you that your manuscript has been judged scientifically suitable for publication and will be formally accepted for publication once it meets all outstanding technical requirements.

Within one week, you’ll receive an e-mail detailing the required amendments. When these have been addressed, you’ll receive a formal acceptance letter and your manuscript will be scheduled for publication.

An invoice will be generated when your article is formally accepted. Please note, if your institution has a publishing partnership with PLOS and your article meets the relevant criteria, all or part of your publication costs will be covered. Please make sure your user information is up-to-date by logging into Editorial Manager at Editorial Manager® and clicking the ‘Update My Information' link at the top of the page. For questions related to billing, please contact billing support.

If your institution or institutions have a press office, please notify them about your upcoming paper to help maximize its impact. If they’ll be preparing press materials, please inform our press team as soon as possible -- no later than 48 hours after receiving the formal acceptance. Your manuscript will remain under strict press embargo until 2 pm Eastern Time on the date of publication. For more information, please contact onepress@plos.org.

Kind regards,

Martin Kieninger

Academic Editor

PLOS One

Additional Editor Comments (optional):

Reviewers' comments:

Reviewer's Responses to Questions

**Comments to the Author**

Reviewer #1: (No Response)

Reviewer #2: All comments have been addressed

**********

2. Is the manuscript technically sound, and do the data support the conclusions?

Reviewer #1: Yes

Reviewer #2: Yes

**********

3. Has the statistical analysis been performed appropriately and rigorously?

Reviewer #1: Yes

Reviewer #2: Yes

**********

4. Have the authors made all data underlying the findings in their manuscript fully available?

The PLOS Data policy

Reviewer #1: Yes

Reviewer #2: Yes

**********

5. Is the manuscript presented in an intelligible fashion and written in standard English?

Reviewer #1: Yes

Reviewer #2: Yes

**********

Reviewer #1: My question regarding the influence of baseline parameter on flow values will be addressed in future studies / publications. I therefore have no further aspects to discuss on this paper.

Reviewer #2: In the revised version the comments and concerns of all reviewers were met by the authors. In my opinion the paper does address an important point in the evaluation of efectiveness of CPR and it improved by adderssing the points of the reviewers.

**********

what does this mean?). If published, this will include your full peer review and any attached files.

If you choose “no”, your identity will remain anonymous but your review may still be made public.

**Do you want your identity to be public for this peer review?**  For information about this choice, including consent withdrawal, please see our Privacy Policy

Reviewer #1: No

Reviewer #2: No

**********

# Editor report: Acceptance letter

**DOI:** 10.1371/journal.pone.0358589.r004

**Authors:** Martin Kieninger

PONE-D-26-13295R1

PLOS One

Dear Dr. Fuchs,

I'm pleased to inform you that your manuscript has been deemed suitable for publication in PLOS One. Congratulations! Your manuscript is now being handed over to our production team.

At this stage, our production department will prepare your paper for publication. This includes ensuring the following:

* All references, tables, and figures are properly cited

* All relevant supporting information is included in the manuscript submission,

* There are no issues that prevent the paper from being properly typeset

You will receive further instructions from the production team, including instructions on how to review your proof when it is ready. Please keep in mind that we are working through a large volume of accepted articles, so please give us a few days to review your paper and let you know the next and final steps.

Lastly, if your institution or institutions have a press office, please let them know about your upcoming paper now to help maximize its impact. If they'll be preparing press materials, please inform our press team within the next 48 hours. Your manuscript will remain under strict press embargo until 2 pm Eastern Time on the date of publication. For more information, please contact onepress@plos.org.

You will receive an invoice from PLOS for your publication fee after your manuscript has reached the completed accept phase. If you receive an email requesting payment before acceptance or for any other service, this may be a phishing scheme. Learn how to identify phishing emails and protect your accounts at https://explore.plos.org/phishing.

If we can help with anything else, please email us at customercare@plos.org.

Thank you for submitting your work to PLOS One and supporting open access.

Kind regards,

PLOS One Editorial Office Staff

on behalf of

Prof. Dr. Martin Kieninger

Academic Editor

PLOS One
